# Automatic image annotation for fluorescent cell nuclei segmentation

**DOI:** 10.1371/journal.pone.0250093

**Published:** 2021-04-16

**Authors:** Fabian Englbrecht, Iris E. Ruider, Andreas R. Bausch

**Affiliations:** 1 Lehrstuhl für Biophysik (E27), Technische Universität München (TUM), Garching, Germany; 2 Center for Protein Assemblies (CPA), Garching, Germany; University of Engineering & Technology, Taxila, PAKISTAN

## Abstract

Dataset annotation is a time and labor-intensive task and an integral requirement for training and testing deep learning models. The segmentation of images in life science microscopy requires annotated image datasets for object detection tasks such as instance segmentation. Although the amount of annotated image data has been steadily reduced due to methods such as data augmentation, the process of manual or semi-automated data annotation is the most labor and cost intensive task in the process of cell nuclei segmentation with deep neural networks. In this work we propose a system to fully automate the annotation process of a custom fluorescent cell nuclei image dataset. By that we are able to reduce nuclei labelling time by up to 99.5%. The output of our system provides high quality training data for machine learning applications to identify the position of cell nuclei in microscopy images. Our experiments have shown that the automatically annotated dataset provides coequal segmentation performance compared to manual data annotation. In addition, we show that our system enables a single workflow from raw data input to desired nuclei segmentation and tracking results without relying on pre-trained models or third-party training datasets for neural networks.

## Introduction

Objection detection becomes of increasing importance in diverse fields of life science microscopy, requiring segmenting, counting or tracking individual cell nuclei. Within the last decade convolutional neural networks (CNNs) have outperformed most object recognition techniques without a deep learning approach [1–3]. With the current deep learning models individual objects of interest such as nuclei of cells are detectable on a pixel level (e. g. instance segmentation) with high precision [4]. Thereby, deep learning methods perform better than traditional cell nuclei detection and segmentation algorithms such as watershed-segmentation [5,6].

In general, segmentation using supervised machine learning techniques requires two steps: data annotation and training. The annotation process is often referred to as data labelling. The labelled data is used together with the associated images as input data to train a machine learning model [5]. Data annotation is often still done manually or can sometimes be semi-automated depending on the morphological complexity of the object and requiring manual fine-tuning [7,8]. This makes supervised machine learning methods a quite labor and time-consuming task. Manually annotating a single object accurately can take several minutes [9]. Therefore, image datasets containing hundreds of objects are mostly partitioned and annotated by several researchers. The annotation inhomogeneity of several humans in return has an impact on the performance of neural networks used for segmentation [10].

On the other hand, the training process of a neural network is a fully automated computational task [11,12]. Due to improvement of graphical hardware and software, the performance of neural networks has steadily increased over the last decades [13]. Despite its advantages, the general applicability of deep learning methods is still hampered by the bottleneck of annotation. This holds especially true in applications of life cell imaging, where often the position and dynamics of individual cells is of interest, which is achieved by nuclei tracing. One common approach is the usage of fully annotated third-party datasets to train a network or using pre-trained models, which thus are not optimized to the imaging modalities of the actual experiments.

**Fig 1:**
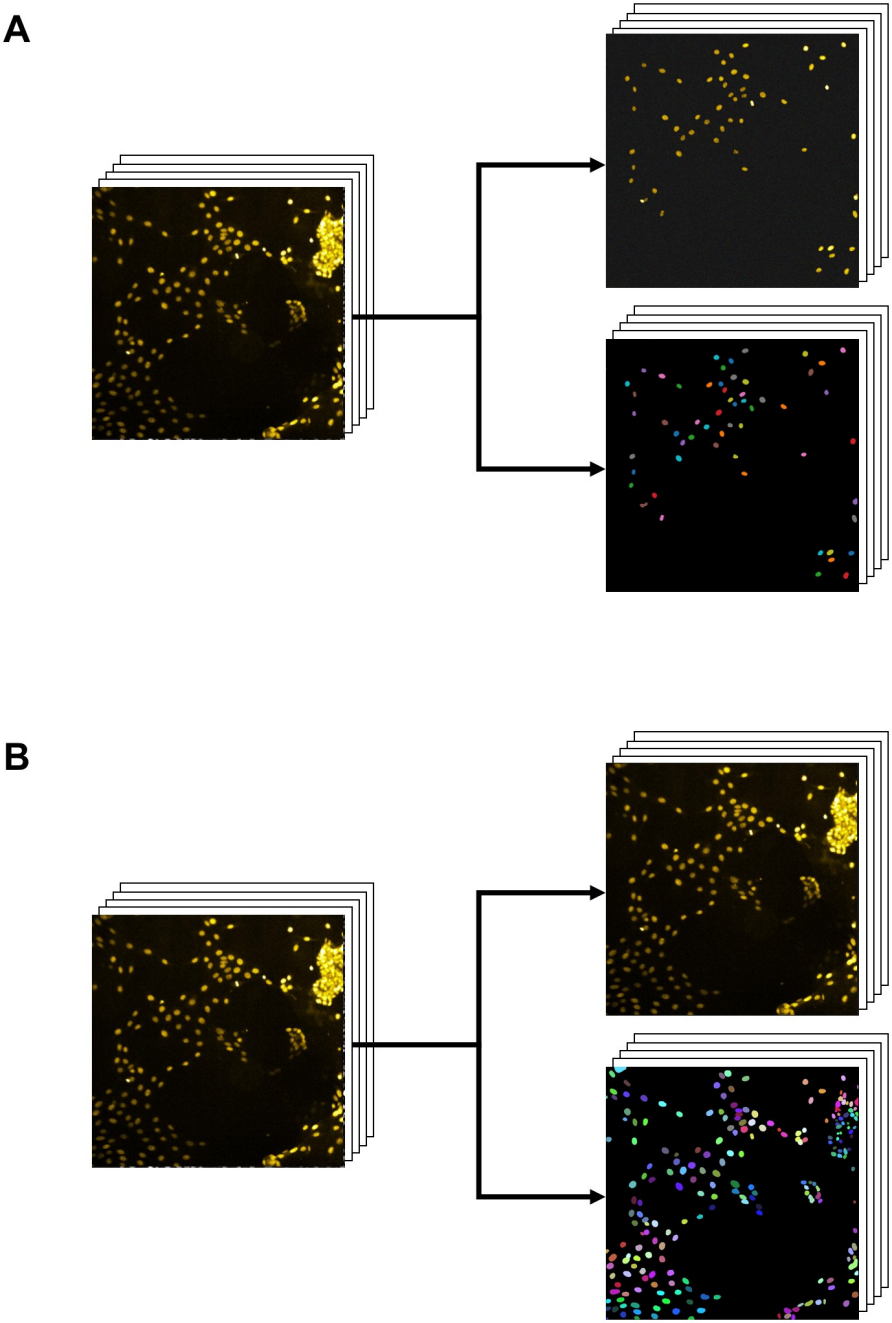
Automated and manual data annotation results. A: Automated nuclei annotation considers a fraction of manually annotated nuclei by coincidently ensuring high precision of labelled nuclei. B: Manual nuclei annotation considers all of the nuclei within the microscopy image. In both cases a training dataset of images and corresponding annotation masks is provided. Human mammary gland epithelial cells (primary cells) are used for demonstration purposes.

Here we demonstrate a method to fully automate the annotation process of a cell nuclei image dataset, which allows flexible adaption of neural networks to specific experimental datasets. Our approach is based on a combination of a pre-processing, a thresholding, a watershed-segmentation, a filtering and a post-processing task applied on a raw image dataset. The output is a dataset of images and masks (annotated data) (Fig 1) which are used as subsequent training dataset for instance segmentation with a convolutional neural network. We demonstrate the accuracy and reliability of the approach by segmenting cell nuclei in fluorescence microscopy images. We also demonstrate the segmentation results in combination with a cell tracking algorithm.

The automated annotation enables to use microscopy image data from individual experiments for providing training datasets, specifically nuclei annotation masks. This is superior to either manual or semi-automated segmentation approaches in terms of time as well as superior to unspecific third-party dataset training approaches (pre-trained models) in terms of specificity and accuracy.

## Materials and methods

**Fig 2:**
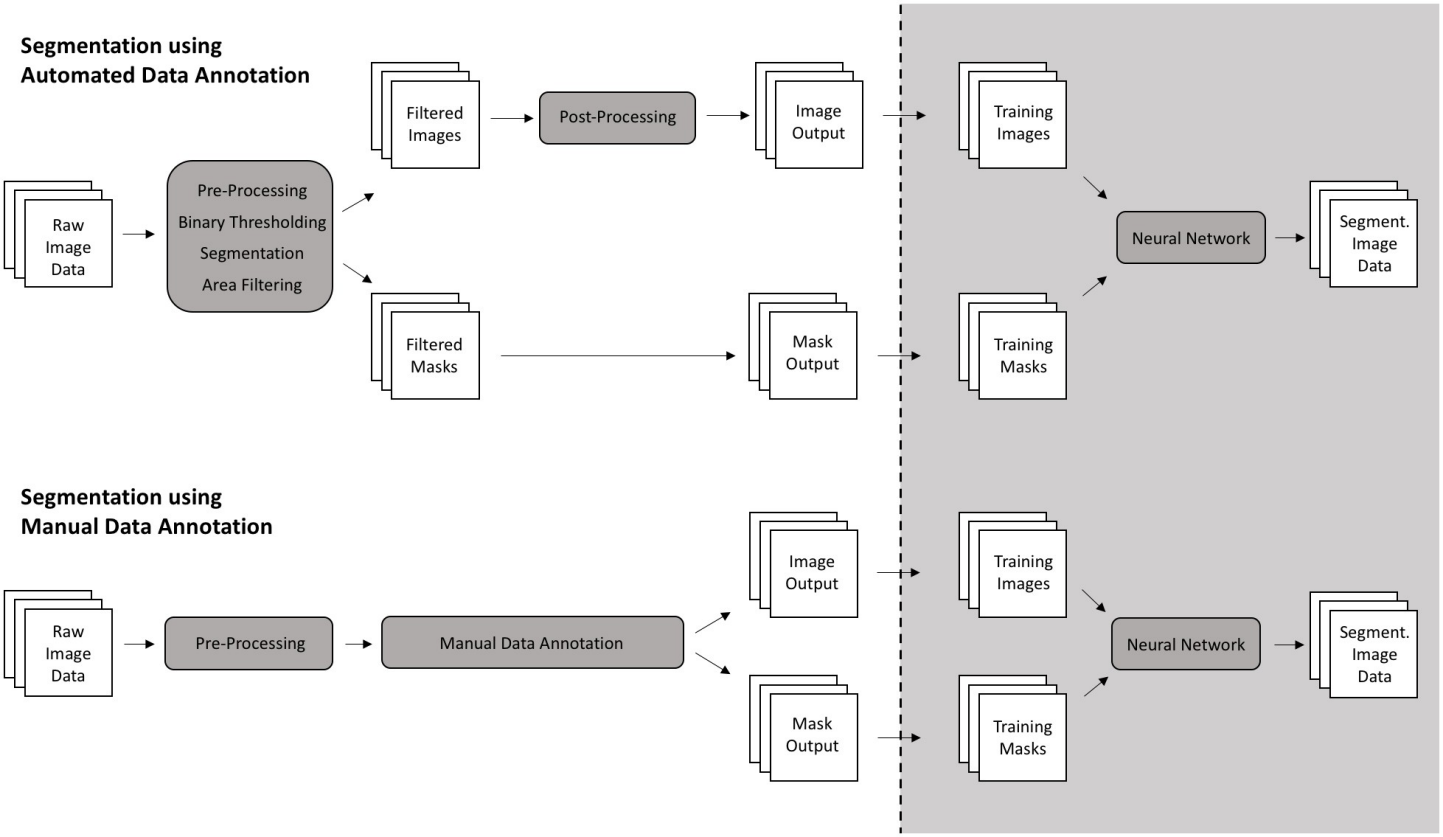
Process of automated and manual nuclei annotation for segmentation with a neural network. Automated data annotation considers image pre-processing, binary thresholding, watershed-segmentation, filtering and post-processing to provide a training dataset for nuclei segmentation with a neural network. Manual data annotation is applied on raw image data (after pre-processing) in order to provide a training dataset of images and annotation masks.

Our automated nuclei annotation method requires several image processing steps (Fig 2). The method integrates these processing steps starting from captured microscopy images and providing annotated data as an output. Key in this process is a combination of image pre-processing, thresholding, watershed-segmentation, filtering and post-processing algorithms.

### Pre-processing

We use conventional image processing techniques for data preparation. These include pixel value conversions, resizing or cropping of the image dataset [14–16] considering the image data input requirements of a convolutional neural network.

### Otsu binarization (thresholding)

Otsu’s method [17] is used for automatic thresholding of binary images in order to separate two object classes in microscopy datasets—specifically fluorescently labelled nuclei from its background [15,16]. The algorithm determines the threshold value that minimizes the intra-class variance, defined as a weighted sum of variances of these two classes:

$$\sigma_{W}^{2}\left(t\right)=W_{0}\left(t\right)\sigma_{0}^{2}\left(t\right)+W_{1}\left(t\right)\sigma_{1}^{2}\left(t\right)$$ (1)

### Watershed segmentation

In a consecutive segmentation step, we apply watershed [18,19]–a widely used segmentation algorithm–to add a label to each individual object separated from the background [15,16,20–22]. These objects include single nuclei, overlapping/touching nuclei, nuclei clusters, nuclei during cell division, as well as image artefacts such as weak fluorescence signal or other artefacts from imaging or culturing cells.

### Pixel area filtering

To filter out objects other than single nuclei from the images, we investigate the pixel area distribution of all objects in our dataset. For identification of the dominant object class in our dataset, specifically single nuclei, we use the median pixel area of all detected objects. A positive and negative threshold is applied in order to exclude objects larger (e.g. cell clusters) or smaller (e.g. cell debris particles, image artefacts) than 50% of the median pixel area [20]. As a result, we receive a dataset of filtered images and annotation masks containing primarily single nuclei with a mean error of including non-single nuclei objects (false positives, false negatives) lower than 1%. Compared to our ground truth dataset the new image dataset contains a total number ratio of 68% single nuclei.

### Post-processing

Cell microscopy images can be viewed as images containing two layers, foreground (cells) and background. On a pixel level, the background is inhomogeneous (even if it looks homogeneous by human eye). By adding random noise to the image dataset, we ensure to add inhomogeneity to the homogeneous black-pixel background [15,16,23]. This results in an improved segmentation performance of the neural network on the corresponding test dataset [24].

### Neural network

To compare the segmentation performance of our automated annotated and manually labelled training dataset we used *StarDist* [25], a convolutional neural network for segmenting star-convex objects such as cell nuclei. The network is based on U-Net [5], a well-established neural network used for high performance cell segmentation and detection tasks as shown in the *Cell Tracking Challenge at ISBI 2015* and the *2018 Data Science Bowl* [5,6]. No pre-trained models were used.

#### Evaluation

To evaluate the nuclei segmentation performance, we used identification of object-level errors. Instance segmentation results of our annotation method are compared with the ground truth of the equivalent dataset to compute the intersection over union (IoU) of all nuclei. True positives (TP), false positives (FP), true negatives (TN) and false negatives (FN). A minimum IoU threshold t (50% of ground truth) was selected to identify correctly segmented objects and any other predicted segmentation mask below the threshold was considered an error. With all TP, FP, TN and FN, a confusion matrix can be determined. Test accuracy parameters Precision (P(t), positive predictive value), Recall (R(t), sensitivity) and F1 score (F1(t), harmonic mean of precision and recall) are computed as follows:

$$P\left(t\right)=\frac{TP(t)}{TP\left(t\right)+FP\left(t\right)}$$ (2)

$$R\left(t\right)=\frac{TP(t)}{TP\left(t\right)+FN\left(t\right)}$$ (3)

$$F1\left(t\right)=\frac{TP(t)}{TP\left(t\right)+\frac{1}{2}(FP\left(t\right)+FN\left(t\right))}$$ (4)

#### Image modalities & dataset

The dataset contains 2D images of fluorescently stained nuclei. Widefield microscopy (*Leica THUNDER Imager)* was used for live cell imaging of Madin-Darby Canine Kidney (MDCK) cells. The cells were stained with SiR-Hoechst, a far-red DNA stain [26]. Cells were incubated with Verapamil to improve fluorescence signal [26]. Videos were acquired with a 20x (0.4 NA) air objective (*Leica*), a frame rate of 10 minutes and a total capture time of 15 hours. The training dataset consists of a total of 5 images containing 6409 nuclei. The test image contains a total of 792 nuclei. All nuclei were annotated manually in order to provide ground truth data. The training and test images were captured at different positions on the same sample. 16-Bit images with a 1:1 aspect ratio and a pixel size of 2048 x 2048 are used.

To prove our method with an individual dataset, we used a subset of 20 training images and 4 test images of the image set BBBC038v1, available from the Broad Bioimage Benchmark Collection [6].

#### Computation

The automated annotation process is computed on a CPU (1.6 GHz Dual-Core Intel Core i5).

For investigating instance segmentation performance, a neural network was trained on a *Nvidia K80* graphics processing unit (GPU). No data augmentation [27,28] was used during the training process. The training process covers a total of 100 training cycles. The network was trained at a batch size of 4 and 256 training steps per epoch, an input patch size of 64, a number of rays for each object of 32, a grid parameter of 2 and an initial learning rate of 0.0003.

## Results

We investigate the performance of a manually annotated microscopy image training dataset compared to our automated data annotation. We specifically compare the annotation and segmentation performances of an automatically and manually annotated training dataset. Furthermore, we want to demonstrate our method on nuclei tracking and large-scale imaging applications.

### Annotation

**Fig 3:**
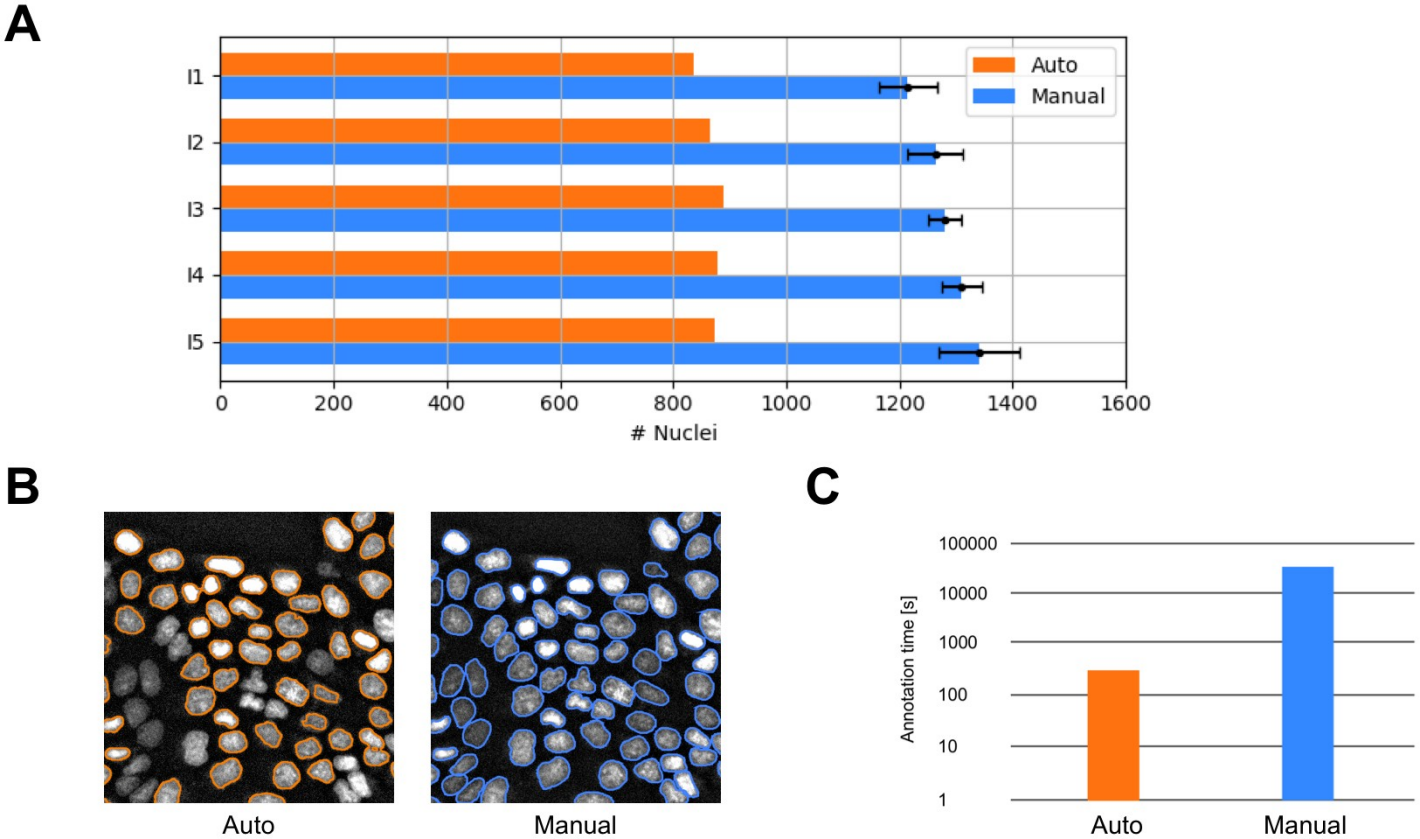
Performance of automated and manual nuclei annotation. A: Number of automatically and manually annotated nuclei for a dataset of five images (I1-I5). B: Regions of interest provided by automatic and manual nuclei annotation. C: Annotation duration of a dataset containing 6409 fluorescently labelled nuclei with automatic (~5 minutes) and manual data annotation (~15 hours).

We first compared the data annotation process of our dataset containing 6409 nuclei between automated and manual labelling. Results are shown in Fig 3 and Table 1.

**Table 1:** Performance of automatically and manually annotated nuclei.

	Automated	Manual
**Number of annotated nuclei**	4339	6409
**Total annotation time [s]**	300	54000
**Annotation time per nucleus [s]**	0.07	8.43
**Mean IoU**	0.790	1.000

Comparing the total number of annotated nuclei of 5 images, manual annotation considers 6409 (100%) nuclei, whereas the automated labelling captures 4339 (67.7%) nuclei.

Whereas manual labelling of the dataset takes 15 hours for a single researcher (total annotation time without considering breaks), automated annotation reduces this process down to a duration of 4:43 minutes (0.5%).

The mean intersection over union (IoU) of automated annotation compared to manual annotation considering all nuclei is 0.545. Considering just the automatically annotated (and filtered) nuclei increases the annotation accuracy to a mean IoU of 0.790 (+44.8%). The F1 score improves from 0.695 to 0.871 (+25.3%). This increase in accuracy is of eminent importance in order to provide a high-quality training dataset for instance segmentation with a neural network.

The significant decrease in time of the annotation process allows our method to be easily applied to larger datasets. We tested our automated annotation on a live cell imaging dataset containing 91 images captured over 15 hours. The dataset contains more than 100,000 nuclei, resulting in an annotation duration of 17:28 minutes.

### Segmentation

**Fig 4:**
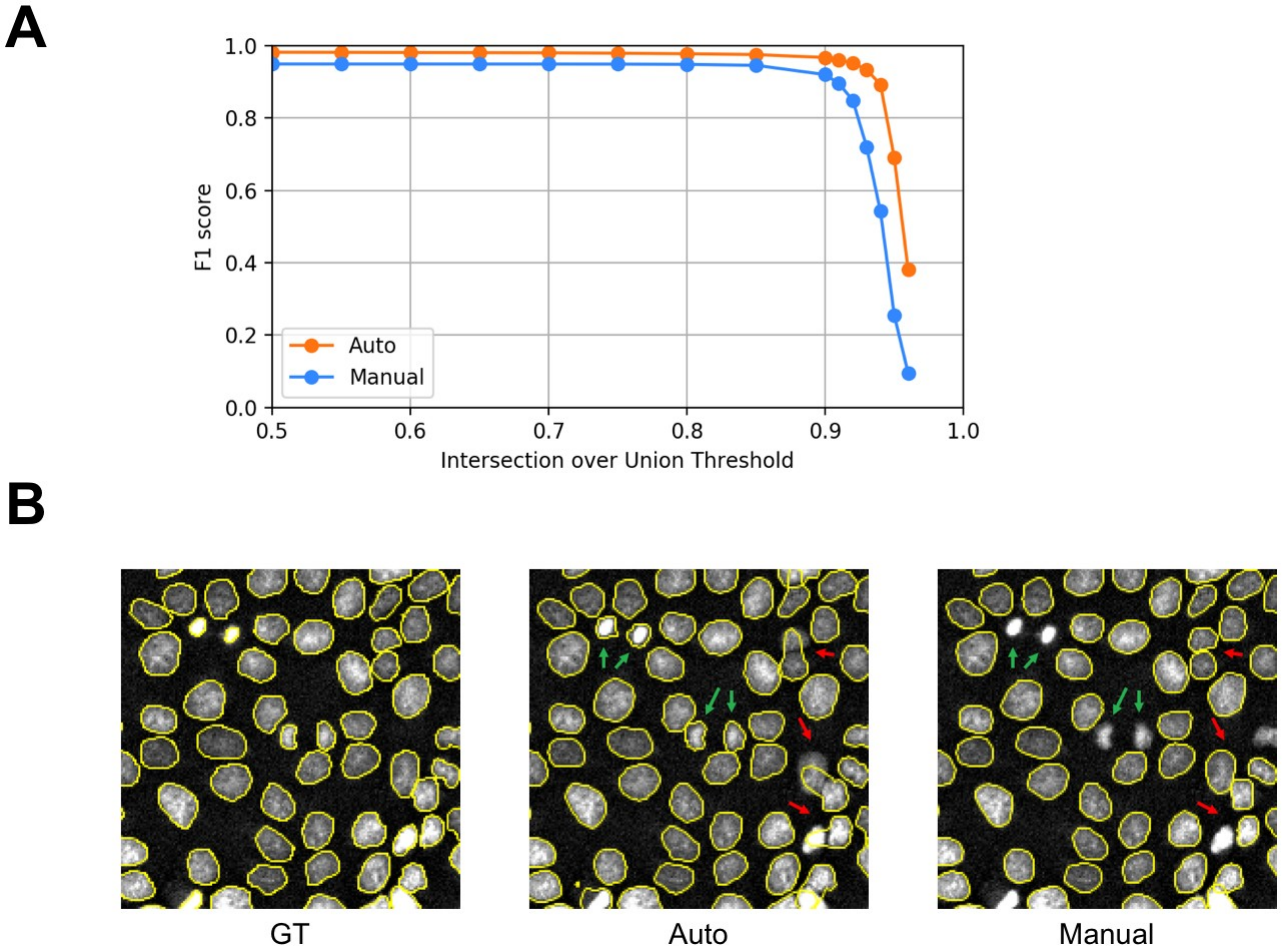
Nuclei segmentation performance of a neural network trained on an automatically and manually annotated training dataset. A: Average F1 score compared to intersection over union of a segmented microscopy dataset trained on automatically and manually annotated nuclei. The average F1 score measures the proportion of correctly segmented objects considering True Positives, False Positives and False Negatives. The intersection over union (IoU) thresholds determine the segmentation accuracy of the neural network (trained on automatically and manually annotated data) compared to the ground truth. High thresholds indicate a strict boundary matching. Average F1 scores remain nearly constant up to IoU = 0.90. At even higher thresholds, accuracy decreases sharply. B: Regions of interest of nuclei segmentation. Ground truth nuclei data are compared to segmentation of a neural network trained on an automatically and manually annotated dataset. Segmentation differences are indicated with green and red arrows. A neural network trained on automatically annotated nuclei provides higher segmentation precision of small nuclei (green arrows). A neural network trained on manually annotated nuclei provides higher accuracy segmenting touching nuclei (red arrows).

We assessed the segmentation performance of our automatically annotated training dataset after training a neural network. The results are compared to a manually annotated training dataset (Fig 4). The neural network trained on automated annotation provides better segmentation of small nuclei (green arrows). Yet, it is only able to detect touching nuclei with low accuracy (red arrows), which are more efficiently segmented by the network trained on ‚the manually annotated data set (red arrows). This is attributed to the fact that touching nuclei are better annotated manually. Both training datasets–automatically and manually annotated–provide coequal overall segmentation performance after training a neural network (Table 2), resulting in a mean IoU of 0.861 (precision: 0.982, recall: 0.984) and 0.863 (precision: 0.999, recall: 0.905). Segmentation results for a long-time measurement (>12 hours) are shown in S1 Video.

**Table 2:** F1 score of segmented nuclei images trained on an automatically and a manually annotated dataset.

	F1 Score	
IoU	Automated	Manual
0.50	0.982	0.949
0.60	0.981	0.949
0.70	0.980	0.949
0.80	0.978	0.948
0.90	0.967	0.920
0.95	0.690	0.255

The sensitivity of nuclei size and shape on our method is unfavorable if the dataset contains nuclei of big size in combination with high nuclei density (touching nuclei) and is favorable if the dataset contains nuclei of small size in combination with low nuclei density. In general, segmenting small nuclei accurately has a higher effect on overall segmentation performance compared to accurately segmenting touching nuclei [29] and should be considered when using our method.

#### Individual datasets

**Fig 5:**
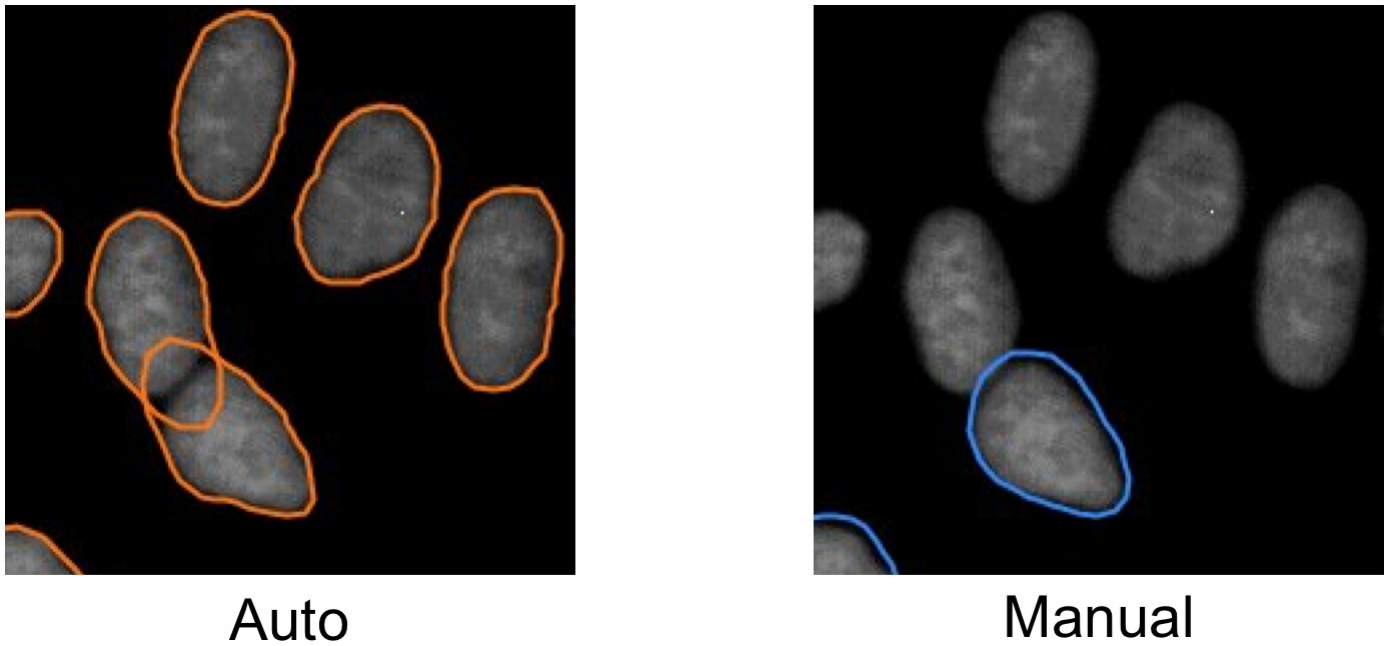
Adaptability of an automatically and manually annotated dataset for nuclei segmentation. Applying automated annotation to an individual dataset and training these data with a neural network provides accurate segmentation results. Nuclei segmentation performs poorly when relying on the training dataset acquired with lower magnifications. To achieve accurate segmentation a time-intensive manual annotation process is required.

We demonstrate the adaptability of our method on a different cell nuclei dataset (Fig 5). The nuclei are imaged with a higher magnification objective (63x) compared to our previous results. Automated annotation on a new image dataset can quickly be performed and results in an accurate segmentation of the test images (mean IoU: 0.792). Results can be provided within minutes. We compare this with the neural network which is trained on the manually annotated dataset at lower magnification (10x), which segments only poorly (mean IoU: 0.155). For better performance a new time-consuming manual annotation of the dataset imaged at higher magnification would be required to train the neural network.

#### Random noise

**Fig 6:**
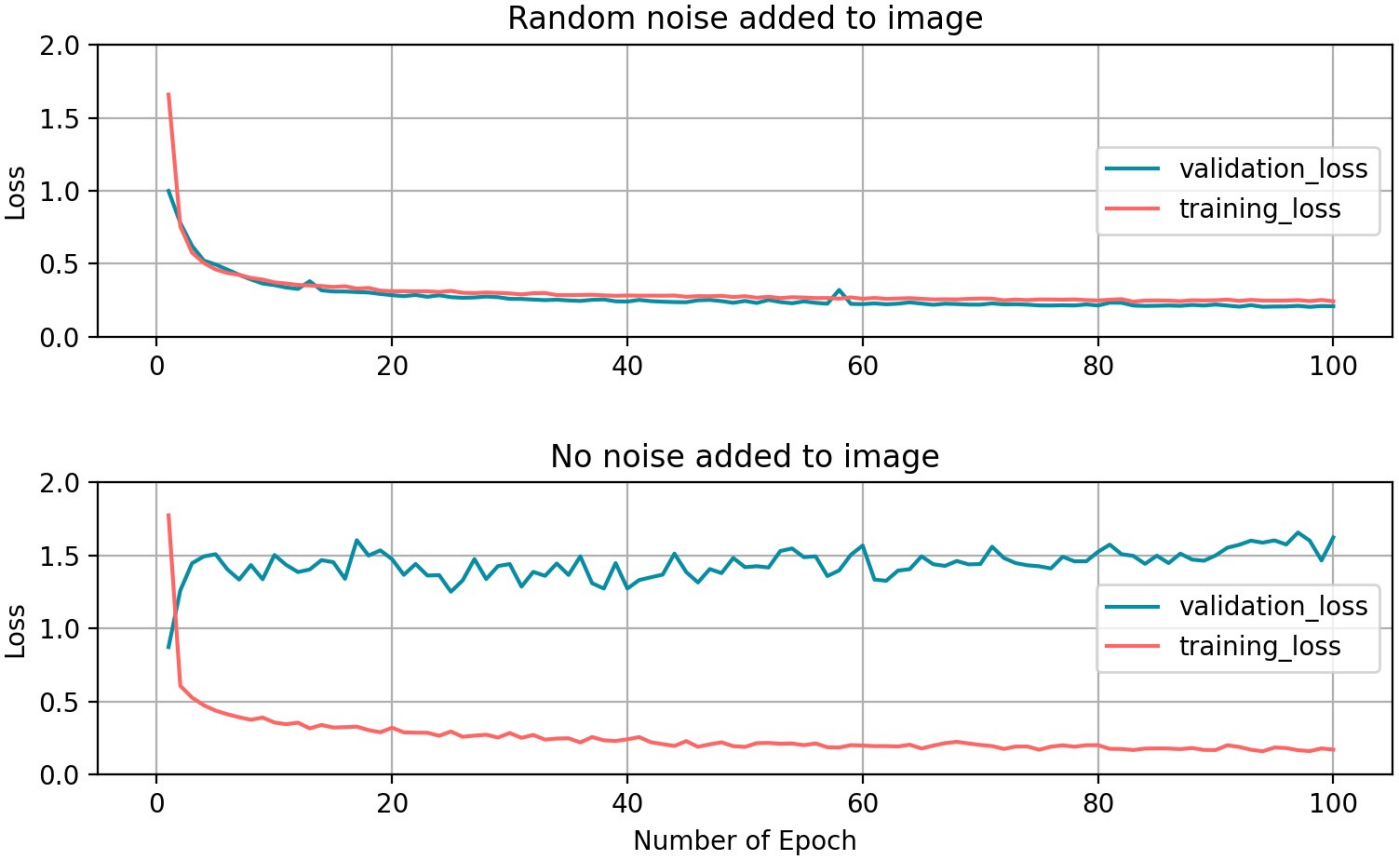
Effect of random noise added to automatically annotated training dataset. Comparison between learning curves of a neural network with and without random noise added to the training dataset. Validation and training loss curves are converging if random noise is added to the automatically annotated training dataset, as the filtered images are without background noise.

Our results show that by adding random noise to the image dataset after pre-processing, the training process is improved (Fig 6) depending on the extent of initial background noise of the original microscopy images before pre-processing. In this case of noise added to the image, training and validation loss curves are converging. Without noise added, the model is fitting to the training data but not all the validation data. In this case the model is not generalizing correctly to a test image dataset.

### Tracking

**Fig 7:**
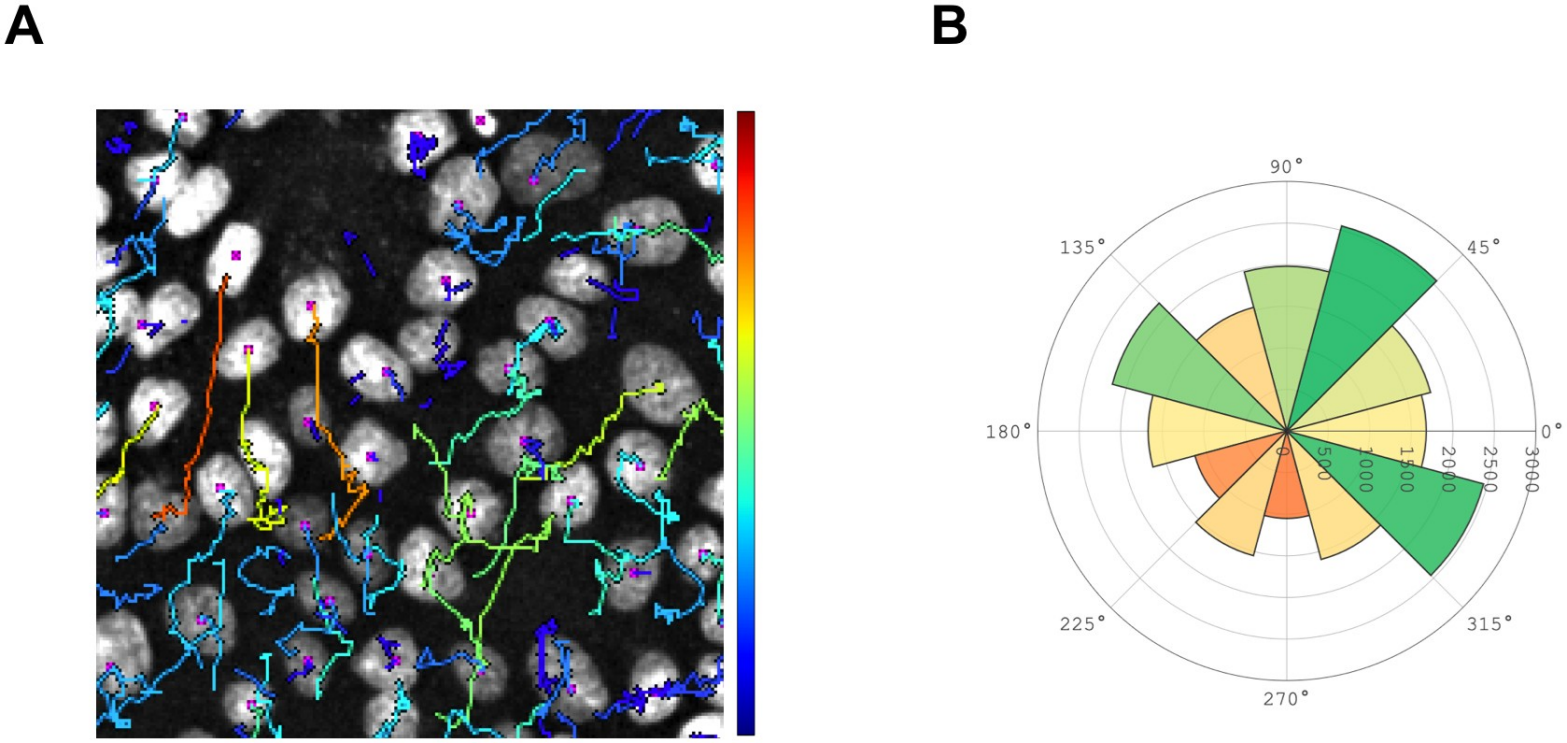
Nuclei tracking results based on an automatically annotated dataset. A: Tracks of fluorescently labelled nuclei. The color of the tracks indicates the speed of each individual nucleus/cell (from blue: Low speed, to red: High speed). B: Mean moving direction of nuclei within 8 hours 20 minutes. Predominant movement directions between 45°-75° and 315°-345° from its starting position.

We use our method for tracking cell nuclei over a timeframe of 8 hours and 20 minutes. After instance segmentation with a neural network, we use *Trackmate* [30], a software widely used for nuclei tracking. *Trackmate* provides reliable tracking of fluorescently labelled nuclei. Results are shown in (Fig 7). Nuclei tracking data such as density, speed, mitosis events, number of tracks or the migration direction of single nuclei can be readily obtained. Long-time tracking (>8 hours) is shown in S2 Video.

## Discussion

For this work we manually annotated a dataset containing 6409 nuclei for providing ground truth. Even during annotation by a single researcher, the amount of decision processes for accurately labelling data–specifically including or excluding areas of interest–is prone to error. This introduces a bias to the dataset that should not be underestimated. Hence, the task of manual data annotation cannot be outsourced for biomedical applications. In fact, most of these datasets are split amongst several researchers for data annotation. A statistical error due to these decision processes amongst multiple annotators has to be considered [10]. The total annotation time of our manually annotated dataset is prohibitive for many applications. Besides, image data annotation is a rather stressful process for the eye which results in errors and enforces continuous breaks between long sets of annotation. Moreover, the mean manual annotation accuracy varied for our experiment depending on the annotation duration. Automated annotation on the other hand provides a highly controllable and consistent way of nuclei annotation. Compared to manual nuclei annotation, automated annotation detects only a fraction (68%) of the total number of nuclei in an image. However, automated annotation can be applied easily to larger datasets. Therefore, the total number of annotated objects can easily be increased which allows to provide high-quality training data for a neural network.

**Fig 8:**
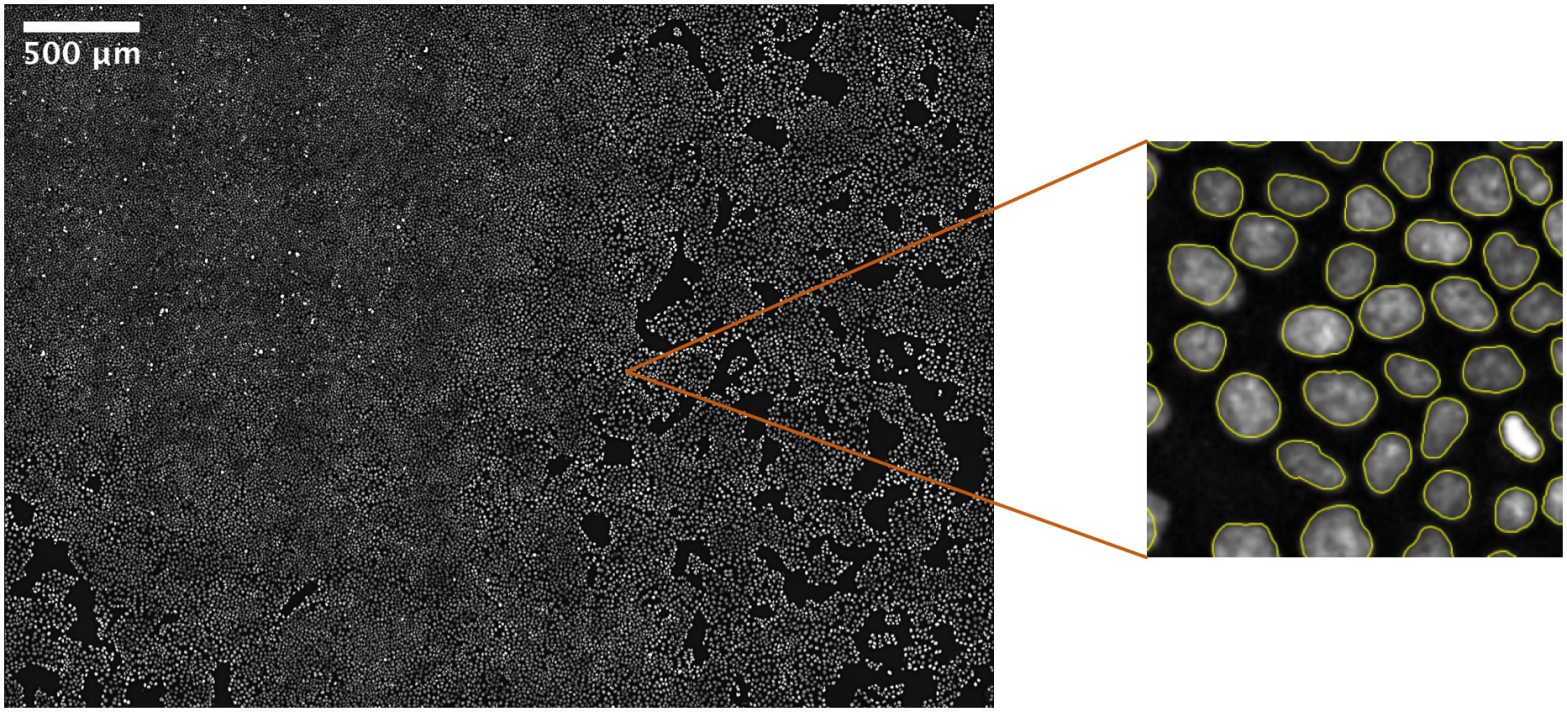
Large scale nuclei segmentation of a widefield microscopy image trained on an automatically annotated dataset. The image has with a view size of 4.2 mm x 3 mm containing more than 60000 single nuclei. Segmentation results are obtained within one hour, including (automatic) annotation and training with a neural network.

To demonstrate the advantage and practicability of the fully automated process, we automatically annotated a large-scale microscopy image of a millimeter range containing more than 60000 nuclei (Fig 8). The nuclei are segmented with a neural network and consequently tracked over 8 hours and 20 minutes with a time resolution of 10 minutes. The whole process including our annotation method on the one hand as well as training the individual dataset on the other hand took less than one hour. The neural network was not pre-trained.

## Conclusions

Our method provides a solution to automate the data annotation process of cell nuclei in microscopy images. The automated annotation process reduces the time and labor-intensive manual data annotation to a minimum and can be adapted to individual datasets. Neural networks which are widely in use for biomedical or clinical applications are used to train on these annotated datasets. Biomedical computer vision tasks such as cell nuclei detection, segmentation or tracking can be analyzed in one single process with high segmentation precision. This allows instant access to experiment results and independency of pre-trained models (transfer-learning) [31] or third-party datasets.

The presented automated annotation is based on the identification of one object class with low variance in area (e.g. single nuclei of cells) and is independent of the object morphology. However, our system works best in combination with a neural network for segmenting star-convex objects, which improves the segmentation of touching nuclei. We see our work to be readily adapted for segmentation of more complex object morphologies, histopathological images, as well as 3D nuclei segmentation applications. Our results demonstrate the effectiveness of automatic nuclei annotation for quantitatively analyzing microscopy time series with high flexibility.

## Supporting information

S1 Video Live cell imaging: Nuclei segmentation. Regions of interest (RoI) after segmentation (yellow). No post-processing is applied to the image data.
(AVI)

S2 Video Live cell imaging: Nuclei tracking. Colored tracks (blue to red) indicate the nuclei velocity at the center of mass (purple circle). No post-processing is applied to the image data.
(AVI)

S1 File Methodical overview of IoU/F1 score enhancement for automatic nuclei annotation. (PDF)

S2 File Sample subset of manual data annotation. (PNG)

S3 File Track of manual data annotation accuracy (sample subset). (PDF)
