# Supplementary material for: Automatic image annotation for fluorescent cell nuclei segmentation
Source: PLoS One. 2021 Apr 16;16(4):e0250093. doi: 10.1371/journal.pone.0250093 (PMC8051811; doi:10.1371/journal.pone.0250093)
Supplement: S1 File — (PDF) [file pone.0250093.s003.pdf]

Automatic Annotation for fluorescent cell nuclei segmentation

Note: Numbers and images represent the whole dataset. The numbers do not represent the single image shown in this overview.

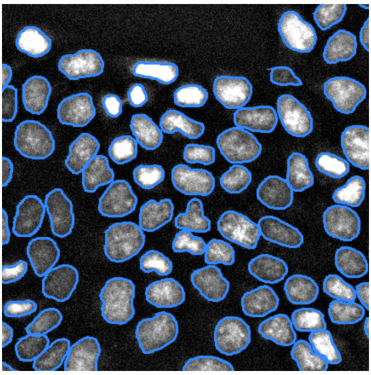

Manual Annotation

ground truth

IoU = 1.000

F1 score = 1.000

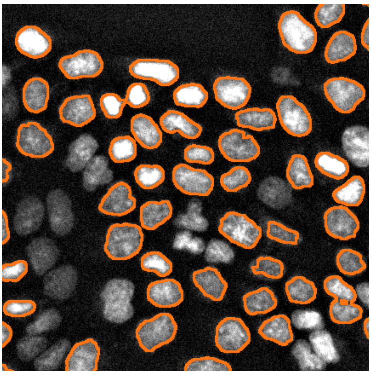

Automatic Annotation

not reduced

IoU = 0.545

F1 score = 0.695

Low IoU / F1 score

Cannot be used as training set for a seg. NN

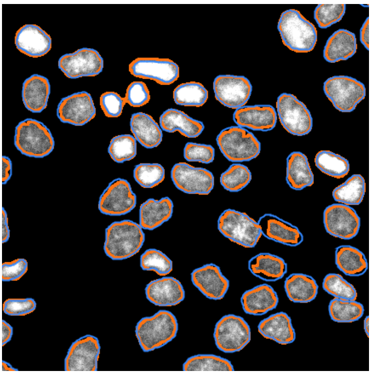

Automatic Annotation

reduced, no noise

IoU = 0.790 (+44.8%) compared to GT (blue label)

F1 score = 0.871 (+25.3%) compared to GT (blue label)

Improved IoU / F1 score

Does not adapt well to the validation / test set

Cannot be used as training set for a seg. NN

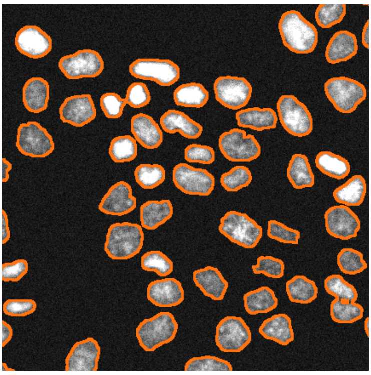

Automatic Annotation

reduced, with random noise

IoU = 0.790

F1 score = 0.871

Adapts well to the validation / test set

Can be used as training set for a seg. NN

Image represents training dataset
