# Supplementary figures and images for: Automatic image annotation for fluorescent cell nuclei segmentation
Source: PLoS One. 2021 Apr 16;16(4):e0250093. doi: 10.1371/journal.pone.0250093 (PMC8051811; doi:10.1371/journal.pone.0250093)

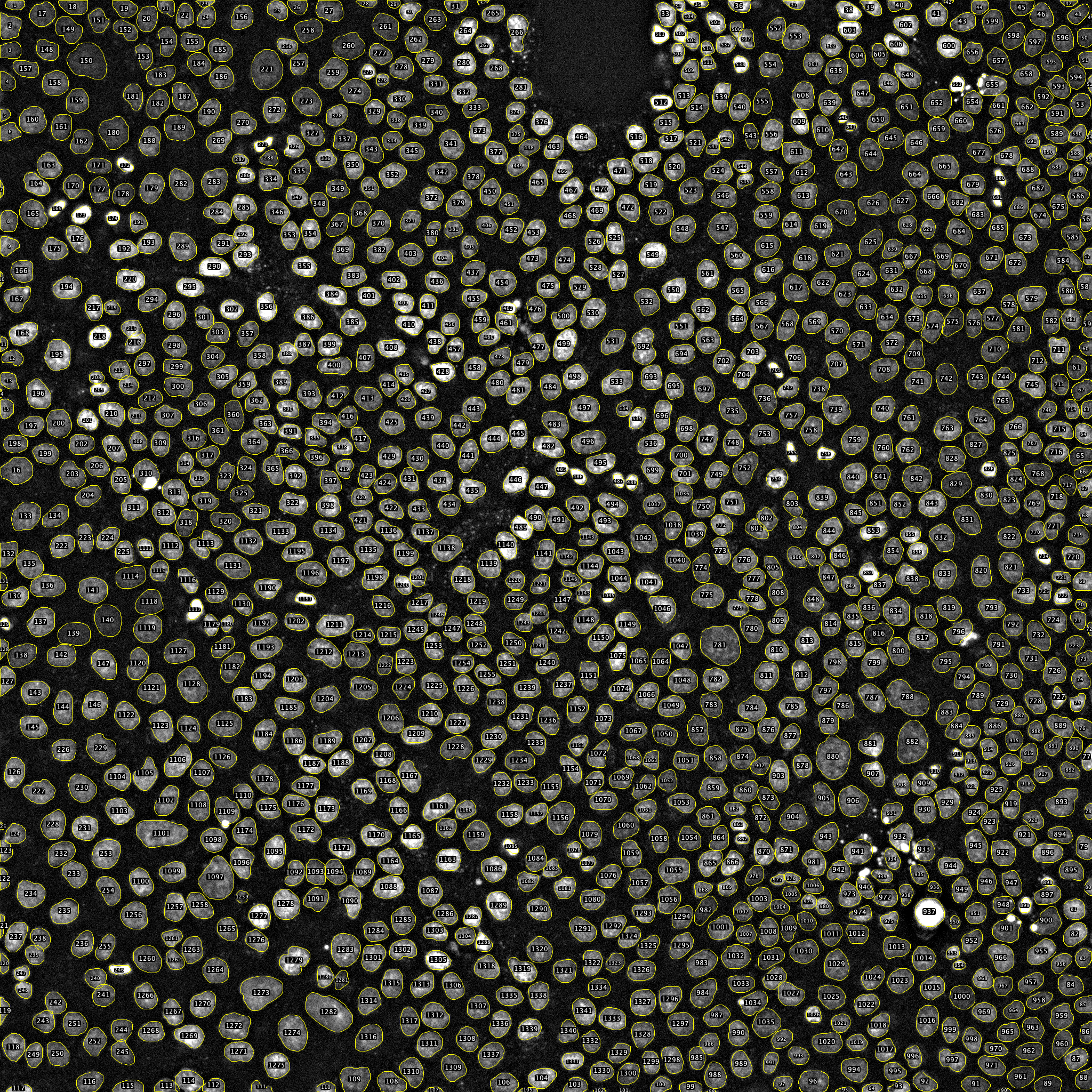

Supplement: S2 File — (PNG) [file pone.0250093.s004.png]
