# Supplementary material for: Automatic image annotation for fluorescent cell nuclei segmentation
Source: PLoS One. 2021 Apr 16;16(4):e0250093. doi: 10.1371/journal.pone.0250093 (PMC8051811; doi:10.1371/journal.pone.0250093)
Supplement: S3 File — (PDF) [file pone.0250093.s005.pdf]

Nucleus ID	Comment
f5#1	
f5#2	
f5#3	
f5#4	
f5#5	
f5#6	
f5#7	
f5#8	
f5#9	
f5#10	
f5#11	
f5#12	
f5#13	
f5#14	
f5#15	
f5#16	
f5#17	
f5#18	
f5#19	
f5#20	
f5#21	
f5#22	
f5#23	tangent f5#24
f5#24	tangent f5#23
f5#25	
f5#26	
f5#27	
f5#28	
f5#29	
f5#30	
f5#31	
f5#32	
f5#33	
f5#34	
f5#35	
f5#36	
f5#37	
f5#38	
f5#39	
f5#40	
f5#41	
f5#42	
f5#43	

f5#44
f5#45
f5#46
f5#47
f5#48
f5#49
f5#50
f5#51
f5#52
f5#53
f5#54
f5#55
f5#56
f5#57
f5#58
f5#59
f5#60
f5#61
f5#62
f5#63
f5#64
f5#65
f5#66
f5#67
f5#68
f5#69
f5#70
f5#71
f5#72
f5#73
f5#74
f5#75
f5#76
f5#77
f5#78
f5#79
f5#80
f5#81
f5#82
f5#83
f5#84
f5#85
f5#86
f5#87

f5#88	
f5#89	uncertain
f5#90	uncertain
f5#91	
f5#92	
f5#93	
f5#94	
f5#95	uncertain
f5#96	
f5#97	
f5#98	uncertain
f5#99	
f5#100	
f5#101	
f5#102	
f5#103	
f5#104	
f5#105	
f5#106	
f5#107	
f5#108	
f5#109	
f5#110	
f5#111	
f5#112	
f5#113	tangent f5#114
f5#114	tangent f5#113
f5#115	
f5#116	
f5#117	
f5#118	
f5#119	
f5#120	
f5#121	
f5#122	
f5#123	tangent f5#124
f5#124	tangent f5#123
f5#125	
f5#126	
f5#127	
f5#128	
f5#129	uncertain
f5#130	
f5#131	uncertain

f5#132	
f5#133	
f5#134	
f5#135	
f5#136	
f5#137	
f5#138	
f5#139	
f5#140	
f5#141	
f5#142	
f5#143	
f5#144	
f5#145	
f5#146	
f5#147	
f5#148	
f5#149	
f5#150	uncertain
f5#151	
f5#152	
f5#153	
f5#154	
f5#155	
f5#156	
f5#157	
f5#158	
f5#159	
f5#160	
f5#161	
f5#162	
f5#163	
f5#164	
f5#165	
f5#166	
f5#167	
f5#168	
f5#169	uncertain
f5#170	
f5#171	
f5#172	uncertain
f5#173	uncertain
f5#174	uncertain
f5#175	

f5#176	
f5#177	
f5#178	
f5#179	
f5#180	uncertain
f5#181	
f5#182	
f5#183	
f5#184	
f5#185	
f5#186	
f5#187	
f5#188	
f5#189	
f5#190	
f5#191	
f5#192	tangent f5#193
f5#193	tangent f5#192
f5#194	
f5#195	
f5#196	
f5#197	
f5#198	
f5#199	
f5#200	
f5#201	
f5#202	
f5#203	
f5#204	
f5#205	
f5#206	
f5#207	
f5#208	tangent f5#209
f5#209	tangent f5#208
f5#210	
f5#211	
f5#212	
f5#213	tangent f5#214
f5#214	tangent f5#213
f5#215	tangent f5#216
f5#216	tangent f5#215
f5#217	
f5#218	
f5#219	

f5#220	
f5#221	
f5#222	
f5#223	
f5#224	
f5#225	
f5#226	
f5#227	
f5#228	
f5#229	
f5#230	
f5#231	
f5#232	
f5#233	
f5#234	
f5#235	
f5#236	
f5#237	
f5#238	tangent f5#239
f5#239	tangent f5#238
f5#240	
f5#241	tangent f5#249
f5#242	tangent f5#243
f5#243	tangent f5#242
f5#244	tangent f5#245
f5#245	tangent f5#244
f5#246	uncertain
f5#247	
f5#248	
f5#249	tangent f5#241
f5#250	
f5#251	
f5#252	
f5#253	
f5#254	
f5#255	
f5#256	
f5#257	
f5#258	
f5#259	
f5#260	
f5#261	
f5#262	
f5#263	

f5#264	
f5#265	
f5#266	uncertain
f5#267	
f5#268	
f5#269	
f5#270	
f5#271	uncertain
f5#272	
f5#273	
f5#274	
f5#275	
f5#276	
f5#277	
f5#278	
f5#279	
f5#280	
f5#281	
f5#282	
f5#283	
f5#284	
f5#285	
f5#286	
f5#287	
f5#288	uncertain
f5#289	
f5#290	
f5#291	tangent f5#292
f5#292	tangent f5#291
f5#293	
f5#294	
f5#295	
f5#296	
f5#297	
f5#298	
f5#299	
f5#300	
f5#301	
f5#302	
f5#303	
f5#304	
f5#305	
f5#306	
f5#307	

f5#308	
f5#309	
f5#310	uncertain
f5#311	
f5#312	
f5#313	
f5#314	
f5#315	
f5#316	
f5#317	
f5#318	
f5#319	
f5#320	
f5#321	
f5#322	
f5#323	
f5#324	
f5#325	
f5#326	
f5#327	
f5#328	
f5#329	
f5#330	
f5#331	
f5#332	
f5#333	
f5#334	
f5#335	
f5#336	
f5#337	
f5#338	
f5#339	
f5#340	
f5#341	
f5#342	
f5#343	
f5#344	
f5#345	
f5#346	
f5#347	
f5#348	
f5#349	
f5#350	
f5#351	

f5#352	
f5#353	
f5#354	
f5#355	
f5#356	
f5#357	
f5#358	
f5#359	
f5#360	
f5#361	
f5#362	
f5#363	
f5#364	
f5#365	tangent f5#366
f5#366	tangent f5#365
f5#367	
f5#368	
f5#369	
f5#370	
f5#371	
f5#372	
f5#373	
f5#374	
f5#375	
f5#376	
f5#377	
f5#378	
f5#379	
f5#380	
f5#381	
f5#382	
f5#383	
f5#384	
f5#385	
f5#386	
f5#387	
f5#388	
f5#389	
f5#390	
f5#391	
f5#392	
f5#393	
f5#394	
f5#395	

f5#396	
f5#397	
f5#398	
f5#399	
f5#400	uncertain
f5#401	
f5#402	
f5#403	
f5#404	
f5#405	
f5#406	
f5#407	
f5#408	
f5#409	
f5#410	
f5#411	
f5#412	
f5#413	
f5#414	
f5#415	
f5#416	
f5#417	
f5#418	
f5#419	
f5#420	
f5#421	
f5#422	
f5#423	
f5#424	
f5#425	
f5#426	
f5#427	
f5#428	uncertain
f5#429	
f5#430	
f5#431	
f5#432	
f5#433	
f5#434	
f5#435	
f5#436	
f5#437	
f5#438	
f5#439	

f5#440	
f5#441	
f5#442	
f5#443	
f5#444	
f5#445	
f5#446	
f5#447	
f5#448	
f5#449	
f5#450	
f5#451	
f5#452	
f5#453	
f5#454	
f5#455	
f5#456	
f5#457	
f5#458	
f5#459	
f5#460	
f5#461	uncertain
f5#462	uncertain
f5#463	
f5#464	
f5#465	
f5#466	
f5#467	
f5#468	
f5#469	
f5#470	
f5#471	
f5#472	
f5#473	
f5#474	
f5#475	
f5#476	
f5#477	
f5#478	
f5#479	
f5#480	
f5#481	
f5#482	
f5#483	

f5#484	
f5#485	uncertain
f5#486	uncertain
f5#487	
f5#488	
f5#489	tangent f5#490
f5#490	tangent f5#489
f5#491	
f5#492	
f5#493	
f5#494	
f5#495	
f5#496	
f5#497	
f5#498	
f5#499	
f5#500	
f5#501	
f5#502	tangent f5#503
f5#503	tangent f5#502
f5#504	
f5#505	
f5#506	tangent f5#507
f5#507	tangent f5#506
f5#508	
f5#509	
f5#510	tangent f5#511
f5#511	tangent f5#510
f5#512	
f5#513	
f5#514	
f5#515	
f5#516	
f5#517	
f5#518	
f5#519	
f5#520	
f5#521	
f5#522	
f5#523	
f5#524	
f5#525	
f5#526	
f5#527	

f5#528	
f5#529	
f5#530	
f5#531	
f5#532	
f5#533	
f5#534	
f5#535	
f5#536	
f5#537	
f5#538	
f5#539	
f5#540	
f5#541	
f5#542	
f5#543	
f5#544	
f5#545	
f5#546	
f5#547	
f5#548	
f5#549	
f5#550	
f5#551	
f5#552	tangent f5#553
f5#553	tangent f5#552
f5#554	
f5#555	
f5#556	
f5#557	
f5#558	
f5#559	
f5#560	
f5#561	
f5#562	
f5#563	
f5#564	
f5#565	
f5#566	
f5#567	
f5#568	
f5#569	
f5#570	
f5#571	

f5#572	
f5#573	
f5#574	tangent f5#575
f5#575	tangent f5#574
f5#576	
f5#577	
f5#578	
f5#579	
f5#580	
f5#581	
f5#582	tangent f5#583
f5#583	tangent f5#582
f5#584	uncertain
f5#585	
f5#586	
f5#587	
f5#588	
f5#589	tangent f5#590
f5#590	tangent f5#589
f5#591	
f5#592	
f5#593	
f5#594	
f5#595	
f5#596	
f5#597	
f5#598	
f5#599	
f5#600	
f5#601	
f5#602	
f5#603	
f5#604	
f5#605	
f5#606	
f5#607	
f5#608	
f5#609	
f5#610	
f5#611	
f5#612	
f5#613	
f5#614	
f5#615	

f5#616	uncertain
f5#617	
f5#618	
f5#619	
f5#620	
f5#621	
f5#622	
f5#623	
f5#624	
f5#625	
f5#626	
f5#627	
f5#628	
f5#629	
f5#630	
f5#631	
f5#632	
f5#633	
f5#634	
f5#635	
f5#636	
f5#637	
f5#638	
f5#639	
f5#640	uncertain
f5#641	uncertain
f5#642	
f5#643	
f5#644	
f5#645	
f5#646	
f5#647	uncertain
f5#648	
f5#649	
f5#650	
f5#651	
f5#652	
f5#653	uncertain
f5#654	uncertain
f5#655	uncertain
f5#656	
f5#657	
f5#658	
f5#659	

f5#660	
f5#661	
f5#662	
f5#663	
f5#664	
f5#665	
f5#666	
f5#667	
f5#668	
f5#669	
f5#670	
f5#671	
f5#672	
f5#673	
f5#674	
f5#675	
f5#676	
f5#677	
f5#678	
f5#679	
f5#680	uncertain
f5#681	uncertain
f5#682	
f5#683	
f5#684	
f5#685	
f5#686	
f5#687	
f5#688	
f5#689	
f5#690	
f5#691	
f5#692	
f5#693	
f5#694	
f5#695	
f5#696	
f5#697	
f5#698	
f5#699	
f5#700	tangent f5#701
f5#701	tangent f5#700
f5#702	
f5#703	

f5#704	
f5#705	uncertain
f5#706	
f5#707	
f5#708	
f5#709	
f5#710	
f5#711	
f5#712	
f5#713	
f5#714	
f5#715	uncertain
f5#716	
f5#717	
f5#718	
f5#719	
f5#720	
f5#721	
f5#722	
f5#723	
f5#724	
f5#725	
f5#726	
f5#727	
f5#728	
f5#729	
f5#730	
f5#731	
f5#732	
f5#733	
f5#734	uncertain
f5#735	
f5#736	
f5#737	
f5#738	
f5#739	
f5#740	
f5#741	
f5#742	
f5#743	
f5#744	
f5#745	uncertain
f5#746	
f5#747	

f5#748	
f5#749	
f5#750	
f5#751	
f5#752	
f5#753	
f5#754	
f5#755	uncertain
f5#756	uncertain
f5#757	
f5#758	
f5#759	
f5#760	
f5#761	
f5#762	
f5#763	
f5#764	
f5#765	
f5#766	
f5#767	
f5#768	
f5#769	
f5#770	tangent f5#771
f5#771	tangent f5#770
f5#772	
f5#773	
f5#774	
f5#775	
f5#776	
f5#777	
f5#778	
f5#779	
f5#780	
f5#781	
f5#782	
f5#783	
f5#784	
f5#785	
f5#786	
f5#787	
f5#788	uncertain
f5#789	
f5#790	
f5#791	

f5#792	
f5#793	
f5#794	
f5#795	
f5#796	uncertain
f5#797	
f5#798	
f5#799	
f5#800	
f5#801	tangent f5#802
f5#802	tangent f5#801
f5#803	
f5#804	
f5#805	
f5#806	
f5#807	
f5#808	
f5#809	
f5#810	
f5#811	
f5#812	
f5#813	
f5#814	
f5#815	
f5#816	uncertain
f5#817	
f5#818	
f5#819	
f5#820	
f5#821	
f5#822	
f5#823	
f5#824	
f5#825	
f5#826	uncertain
f5#827	
f5#828	
f5#829	
f5#830	
f5#831	
f5#832	
f5#833	
f5#834	
f5#835	

f5#836	
f5#837	
f5#838	uncertain
f5#839	
f5#840	
f5#841	
f5#842	
f5#843	
f5#844	
f5#845	
f5#846	
f5#847	
f5#848	
f5#849	
f5#850	uncertain
f5#851	
f5#852	
f5#853	uncertain
f5#854	
f5#855	
f5#856	
f5#857	
f5#858	
f5#859	
f5#860	
f5#861	
f5#862	
f5#863	
f5#864	
f5#865	
f5#866	
f5#867	
f5#868	
f5#869	
f5#870	
f5#871	
f5#872	
f5#873	
f5#874	
f5#875	
f5#876	
f5#877	
f5#878	
f5#879	

f5#880	
f5#881	
f5#882	
f5#883	
f5#884	
f5#885	
f5#886	
f5#887	
f5#888	
f5#889	
f5#890	
f5#891	
f5#892	
f5#893	uncertain
f5#894	
f5#895	
f5#896	
f5#897	
f5#898	uncertain
f5#899	uncertain
f5#900	uncertain
f5#901	uncertain
f5#902	
f5#903	
f5#904	
f5#905	
f5#906	
f5#907	
f5#908	
f5#909	
f5#910	uncertain
f5#911	uncertain
f5#912	
f5#913	
f5#914	
f5#915	
f5#916	
f5#917	
f5#918	
f5#919	
f5#920	
f5#921	
f5#922	
f5#923	

f5#924	
f5#925	
f5#926	
f5#927	
f5#928	
f5#929	
f5#930	
f5#931	uncertain
f5#932	uncertain
f5#933	uncertain
f5#934	
f5#935	
f5#936	uncertain
f5#937	uncertain
f5#938	
f5#939	
f5#940	uncertain
f5#941	
f5#942	
f5#943	
f5#944	
f5#945	
f5#946	
f5#947	
f5#948	
f5#949	
f5#950	
f5#951	
f5#952	
f5#953	
f5#954	
f5#955	
f5#956	
f5#957	
f5#958	
f5#959	
f5#960	
f5#961	
f5#962	
f5#963	
f5#964	
f5#965	
f5#966	
f5#967	

f5#968	
f5#969	
f5#970	
f5#971	
f5#972	
f5#973	
f5#974	
f5#975	
f5#976	
f5#977	tangent f5#978
f5#978	tangent f5#977
f5#979	tangent f5#980
f5#980	tangent f5#979
f5#981	
f5#982	
f5#983	
f5#984	
f5#985	tangent f5#986
f5#986	tangent f5#985
f5#987	
f5#988	
f5#989	
f5#990	
f5#991	
f5#992	
f5#993	
f5#994	
f5#995	
f5#996	
f5#997	uncertain
f5#998	
f5#999	
f5#1000	
f5#1001	
f5#1002	
f5#1003	
f5#1004	
f5#1005	
f5#1006	
f5#1007	
f5#1008	
f5#1009	
f5#1010	
f5#1011	

f5#1012	
f5#1013	
f5#1014	
f5#1015	
f5#1016	
f5#1017	
f5#1018	
f5#1019	
f5#1020	
f5#1021	
f5#1022	
f5#1023	
f5#1024	
f5#1025	
f5#1026	uncertain
f5#1027	tangent f5#1028
f5#1028	tangent f5#1027
f5#1029	
f5#1030	
f5#1031	
f5#1032	
f5#1033	
f5#1034	
f5#1035	
f5#1036	
f5#1037	
f5#1038	
f5#1039	
f5#1040	
f5#1041	
f5#1042	
f5#1043	
f5#1044	
f5#1045	uncertain
f5#1046	
f5#1047	
f5#1048	
f5#1049	
f5#1050	
f5#1051	
f5#1052	
f5#1053	
f5#1054	
f5#1055	

f5#1056	
f5#1057	
f5#1058	
f5#1059	
f5#1060	
f5#1061	
f5#1062	
f5#1063	
f5#1064	
f5#1065	
f5#1066	
f5#1067	
f5#1068	
f5#1069	
f5#1070	
f5#1071	
f5#1072	
f5#1073	
f5#1074	
f5#1075	
f5#1076	
f5#1077	
f5#1078	
f5#1079	
f5#1080	
f5#1081	
f5#1082	
f5#1083	tangent f5#1084
f5#1084	tangent f5#1083
f5#1085	uncertain
f5#1086	
f5#1087	
f5#1088	
f5#1089	
f5#1090	
f5#1091	
f5#1092	
f5#1093	tangent f5#1094
f5#1094	tangent f5#1093
f5#1095	
f5#1096	
f5#1097	
f5#1098	
f5#1099	

f5#1100	
f5#1101	
f5#1102	
f5#1103	
f5#1104	
f5#1105	
f5#1106	
f5#1107	
f5#1108	
f5#1109	
f5#1110	
f5#1111	
f5#1112	
f5#1113	uncertain
f5#1114	
f5#1115	
f5#1116	
f5#1117	uncertain
f5#1118	
f5#1119	
f5#1120	
f5#1121	
f5#1122	
f5#1123	
f5#1124	
f5#1125	
f5#1126	
f5#1127	
f5#1128	
f5#1129	
f5#1130	
f5#1131	
f5#1132	
f5#1133	
f5#1134	
f5#1135	
f5#1136	uncertain
f5#1137	
f5#1138	
f5#1139	
f5#1140	
f5#1141	
f5#1142	
f5#1143	

f5#1144
f5#1145
f5#1146
f5#1147
f5#1148
f5#1149
f5#1150
f5#1151
f5#1152
f5#1153
f5#1154
f5#1155
f5#1156
f5#1157
f5#1158
f5#1159
f5#1160
f5#1161
f5#1162
f5#1163
f5#1164
f5#1165
f5#1166
f5#1167
f5#1168
f5#1169
f5#1170
f5#1171
f5#1172
f5#1173
f5#1174
f5#1175
f5#1176
f5#1177
f5#1178
f5#1179
f5#1180
f5#1181
f5#1182
f5#1183
f5#1184
f5#1185
f5#1186
f5#1187

f5#1188	
f5#1189	
f5#1190	
f5#1191	uncertain
f5#1192	
f5#1193	
f5#1194	
f5#1195	
f5#1196	
f5#1197	
f5#1198	
f5#1199	
f5#1200	
f5#1201	
f5#1202	
f5#1203	
f5#1204	
f5#1205	
f5#1206	
f5#1207	
f5#1208	
f5#1209	
f5#1210	
f5#1211	
f5#1212	
f5#1213	
f5#1214	
f5#1215	
f5#1216	
f5#1217	
f5#1218	
f5#1219	
f5#1220	
f5#1221	
f5#1222	
f5#1223	
f5#1224	
f5#1225	
f5#1226	
f5#1227	
f5#1228	
f5#1229	
f5#1230	
f5#1231	

f5#1232	
f5#1233	
f5#1234	
f5#1235	
f5#1236	
f5#1237	
f5#1238	
f5#1239	
f5#1240	
f5#1241	
f5#1242	
f5#1243	tangent f5#1244
f5#1244	tangent f5#1243
f5#1245	
f5#1246	
f5#1247	
f5#1248	
f5#1249	
f5#1250	
f5#1251	
f5#1252	
f5#1253	
f5#1254	
f5#1255	
f5#1256	
f5#1257	
f5#1258	
f5#1259	uncertain
f5#1260	
f5#1261	
f5#1262	
f5#1263	
f5#1264	
f5#1265	
f5#1266	
f5#1267	
f5#1268	
f5#1269	
f5#1270	
f5#1271	
f5#1272	
f5#1273	
f5#1274	
f5#1275	

f5#1276	
f5#1277	
f5#1278	
f5#1279	uncertain
f5#1280	
f5#1281	uncertain
f5#1282	
f5#1283	
f5#1284	
f5#1285	
f5#1286	
f5#1287	
f5#1288	
f5#1289	
f5#1290	
f5#1291	
f5#1292	
f5#1293	
f5#1294	
f5#1295	
f5#1296	
f5#1297	
f5#1298	
f5#1299	
f5#1300	
f5#1301	
f5#1302	
f5#1303	
f5#1304	
f5#1305	
f5#1306	
f5#1307	
f5#1308	
f5#1309	
f5#1310	
f5#1311	
f5#1312	
f5#1313	
f5#1314	
f5#1315	
f5#1316	
f5#1317	
f5#1318	
f5#1319	uncertain

f5#1320	
f5#1321	
f5#1322	
f5#1323	
f5#1324	
f5#1325	
f5#1326	
f5#1327	
f5#1328	
f5#1329	
f5#1330	
f5#1331	
f5#1332	
f5#1333	
f5#1334	
f5#1335	
f5#1336	
f5#1337	
f5#1338	
f5#1339	
f5#1340	uncertain
f5#1341	
